# Low-Dose Naltrexone Use in Postural Orthostatic Tachycardia Syndrome: A Case Series

**DOI:** 10.7759/cureus.43426

**Published:** 2023-08-13

**Authors:** Samantha J Stallkamp Tidd, Christopher Cantrell, Brady D Greene, Robert Wilson

**Affiliations:** 1 Neurology, Cleveland Clinic Lerner College of Medicine, Cleveland Clinic, Cleveland, USA; 2 Neurology, Cleveland Clinic, Cleveland, USA

**Keywords:** low-dose naltrexone, postural orthostatic tachycardia syndrome (pots), pots treatment, autonomic neurology, autonomic cardiology, autonomic nervous system dysfunction

## Abstract

Introduction

In recent years, low-dose naltrexone has emerged as a novel off-label therapy for many chronic conditions including postural orthostatic tachycardia syndrome (POTS), however, there is little evidence for its efficacy.

Methods

In this institutional review board (IRB)-approved case series, the charts of six tilt table-confirmed patients with POTS who underwent a trial of low-dose naltrexone (LDN) at our institution were reviewed. Medical history, subjective description of symptom severity, the continuation of therapy, tolerability, and scores on patient-reported outcome measures (Patient-Reported Outcomes Measurement Information System {PROMIS} Fatigue, PROMIS physical and mental health, Generalized Anxiety Disorder Assessment {GAD}-7, Patient Health Questionnaire {PHQ}-9, and Composite Autonomic Symptom Score {COMPASS}) were collected at therapy initiation and six to 12 months after the start of LDN.

Results

Three out of six reviewed patients reported an improvement in their POTS after the initiation of LDN. Two patients discontinued the therapy due to a lack of perceived benefit. No side effects or adverse outcomes were reported. The patient-reported outcome measures of PROMIS Fatigue, PROMIS physical and mental health, GAD-7, PHQ-9, and COMPASS showed inconsistent changes over the course of therapy, with some patients showing improvement or stability and others showing worsening. The small sample size and incomplete response rate did not allow for extensive statistical analysis.

Conclusion

As seen in its use in other conditions, LDN appears to have a favorable safety and side effect profile in patients with POTS but has little evidence for efficacy. Although some patients noted benefit, patient-reported outcome measures show a variable response profile. High-quality randomized controlled trials are needed to determine if the treatment is efficacious and should be used outside of a trial basis.

## Introduction

Postural orthostatic tachycardia syndrome (POTS) is a heterogeneous disorder of the autonomic nervous system that is largely characterized by excessive tachycardia and orthostatic intolerance upon standing that improves when supine. Additional symptoms are varied and can include fatigue, palpitations, headaches, chest pain, and gastrointestinal symptoms [1]. POTS can often be disabling, leading to decreased quality of life, unemployment, and income loss [2,3]. Although the true prevalence of POTS is unknown, it is estimated to affect 0.1-1% of the US population [4]. Recently, diagnosed cases are rising with more patients developing the syndrome after infection with coronavirus disease 2019 (COVID-19) [5]. First-line therapy for POTS is non-pharmacological treatment with increased salt intake and hydration, compression garments, and exercise. However, many POTS patients require additional therapies. Despite the increasing prevalence of the disease and the significant impact it has on patients, there are currently no FDA-approved drug treatments for POTS. Clinicians approach pharmacological therapy largely based on societal guidelines and loose recommendations, given the lack of high-quality studies backing any one regimen [6,7]. POTS researchers must discover novel treatment options and, importantly, rigorously evaluate these therapies in high-quality clinical trials [7].

Naltrexone hydrochloride is an FDA-approved drug for the treatment of opioid and alcohol dependence due to its opioid antagonism at dosages ranging from 50-100 mg [8]. In the 1980s, the use of naltrexone at lower dosages (4.5 mg or less), termed “low-dose naltrexone” or “LDN,” began to be explored as a potential anti-inflammatory medication [8,9]. Since then, the popularity of the off-label use of this drug has skyrocketed for a growing list of chronic conditions including chronic pain, complex regional pain syndrome, inflammatory bowel disease, fibromyalgia, rheumatoid arthritis, and neuropathy [8-10]. Although studies have established favorable tolerability and safety of use in many of these conditions, there is sparse evidence for its efficacy [8,9]. Due to the drug’s presumed safety and potential anti-inflammatory properties, it continues to be trialed on an off-label basis in more and more conditions, especially in those with limited evidence-based treatment options, including POTS and long-COVID [11,12]. The use of LDN in POTS has only been described in detail by one prior case report of a woman with comorbid mast cell activation syndrome and small intestinal bacterial overgrowth. This patient noted benefits after undergoing a trial with a drug regimen including LDN, intravenous immunoglobulin (IVIg), and antibiotic therapy. It is unknown if individually one of these therapies was the most beneficial for her POTS, or if the unique cocktail of treatments afforded improvement [11]. Ongoing trials are critically evaluating this drug’s potential utility, including a placebo-controlled clinical trial in POTS patients (ClinicalTrials.gov Identifier: NCT05363514).

Here, we present a case series of patients who trialed LDN as a part of their POTS treatment regimen. We will discuss the potential mechanism of action of LDN in this population and the need for ongoing high-quality clinical trials.

## Materials and methods

Chart review

In this Institutional Review Board (IRB)-approved case series, the charts of patients who received low-dose naltrexone therapy in the autonomic neurology department were reviewed for inclusion in the series. Patients had to meet the following inclusion criteria: (1) have a tilt table-confirmed POTS diagnosis defined by the diagnostic criteria in Table 1 [1]. (2) Taken LDN therapy for a minimum of one month. (3) Returned for a follow-up visit after drug initiation between six months and one year.

**Table 1:** Diagnostic criteria of postural orthostatic tachycardia syndrome (POTS).

POTS diagnostic criteria
A sustained heart rate (HR) increment of not less than 30 beats/minute within 10 minutes of standing or head-up tilt. For individuals who are 12-19 years old, the required HR increment is at least 40 beats/minute.
An absence of orthostatic hypotension (i.e., no sustained systolic blood pressure {BP} drop of 20 mmHg or more).
Frequent symptoms of orthostatic intolerance during standing, with rapid improvement upon return to a supine position. Symptoms may include lightheadedness, palpitations, tremulousness, generalized weakness, blurred vision, and fatigue.
Duration of symptoms for at least three months.
Absence of other conditions explaining sinus tachycardia.

Six patients met this inclusion criteria. Information on demographics, medical history, use of naltrexone, disease courses, examinations, test results, and routine patient-entered questionaries were collected. The start of the collection period was the date of prescription, and the end was the first follow-up visit six to twelve months after therapy initiation plus or minus two months for patient-entered data.

Reviewed questionnaires

The PROMIS Fatigue 10a questionnaire is a 10-question survey that can be used to track fatigue in patients with chronic illness. This score is reported as a t-score with a mean of 50 and a standard deviation of 10. A higher score on this survey corresponds to increased fatigue [13]. The PROMIS physical health score is a composite t-score from the PROMIS Global Health Questionnaire V1.0 that attempts to capture the overall physical well-being of an individual with higher scores on this measure representing a higher level of health. The PROMIS Global Mental Health is a composite t-score from the PROMIS Global Health Questionnaire V1.0 that measures the overall mental well-being of an individual with higher scores representing a higher level of health [14]. Generalized Anxiety Disorder Assessment (GAD)-7 is a frequently used assessment for anxiety screening as well as for monitoring anxiety treatment over time. Score breakdowns of 0-4, 5-9, 10-14, and 15-21 correspond to minimal, mild, moderate, and severe levels of anxiety, respectively [15]. The Patient Health Questionnaire (PHQ-9) is a screening and monitoring tool used for major depression. A score of 10 or more is generally used as the cut-off value for a positive screen [16]. The Composite Autonomic Symptom Score (COMPASS) 31 is a survey used to assess the severity and symptoms of those with autonomic conditions. A higher score on this survey indicates higher severity [17].

## Results

Case descriptions

Patient A

Patient A is a 30-year-old female who was diagnosed with POTS in late 2020 after contracting COVID-19. In addition to standard non-pharmacological management for her POTS, she completed trials of beta-blockers, fludrocortisone, and memantine, but discontinued these therapies due to side effects and continued symptoms. LDN was explored as a potential treatment option due to her additional lower extremity neuropathic pain, which had failed three traditional treatment trials. Two months prior to LDN initiation, a full history and neurological examination were completed. Her most bothersome symptoms were weakness and pain in her legs with exertion, tachycardia, shortness of breath on exertion, and headaches. The patient was started on 1 mg of naltrexone, which was subsequently escalated to 4 mg. During the treatment period, the patient was started on ivabradine in conjunction with her naltrexone therapy. After five months on LDN, the patient sought to discontinue the medication, citing that while it had slightly improved her pain levels at rest, it did not improve her pain with activity, which was her largest reason for starting the therapy. No side effects or adverse events were reported.

Patient B

Patient B is a 54-year-old female with a yearlong history of POTS. In addition to standard non-pharmacological management, she had previously completed trials of beta blockers, fludrocortisone, and midodrine for POTS therapy before discontinuing due to side effects. At the start of LDN treatment, she was taking ivabradine and pyridostigmine for her POTS. LDN was considered as an additional therapy due to the patient's severe neck pain. At the prescribing visit, the patient reported fatigue, neck pain, dry eyes, and dry mouth. Her examination was significant for muscle tightness and limited range of motion of her neck with side bend and rotation. She was started on 1 mg of naltrexone, and her dose was escalated to 2 mg after two months of therapy before being discontinued completely after 71 days of therapy. LDN was discontinued due to a lack of perceived benefit, persistent neck pain, and the desire to try another drug. No adverse side effects were reported.

Patient C

Patient C is a 48-year-old female with a two-year history of POTS. She had previously trialed beta-blockers, pyridostigmine, droxidopa, calcium channel blockers, clonidine, and ivabradine in addition to standard non-pharmacological management with continued symptoms. At the time of therapy initiation, she was taking acebutolol and clonidine for her POTS. LDN was considered due to numerous failed treatment trials and chronic pain. At the prescribing visit, her main symptoms were intermittent bradycardia, fatigue, adrenergic symptoms, and pain, especially around menses. Her examination was notable for gait unsteadiness after 50 feet. Six months after treatment initiation, the patient reported that her symptoms improved on LDN, however, she continued to experience fatigue and pain. Her examination at the end visit was normal.

Patient D

Patient D is a 51-year-old female with a four-year history of POTS. She had previously failed trials with metoprolol due to increased anxiety, fludrocortisone due to weight gain, and pyridostigmine due to nausea. At the time of LDN initiation, she was being treated with ivabradine, duloxetine, gabapentin, and hydrocortisone for her POTS and small fiber neuropathy. LDN was trialed due to foot and leg pain refractory to gabapentin and duloxetine. Six months after treatment initiation, she reported that LDN was helping with her fatigue, headaches, and pain.

Patient E

Patient E is a 31-year-old female with a three-year history of POTS. She previously trialed modafinil, fludrocortisone, and midodrine, but discontinued each due to side effects. At the time of LDN initiation, she was maintained on ivabradine and propranolol. LDN was chosen as an additional therapy due to her fatigue and continued symptoms despite multiple interventions. At the prescribing visit, the patient reported symptoms of lightheadedness, loss of balance, and fatigue. Neurological examination at this time was within normal limits. Five months after starting naltrexone, the patient reported that her most significant symptoms included fatigue, lightheadedness, and intermittent facial drooping, but she did not comment on her experience with LDN.

Patient F

Patient F is a 62-year-old female with a seven-year history of POTS. She previously failed trials of metoprolol, fludrocortisone, and midodrine citing a lack of improvement. LDN was initiated due to continued POTS symptoms and Raynaud's phenomenon. LDN was initiated in addition to her preexisting pyridostigmine and modafinil regimen. At the prescribing visit, she reported symptoms of fatigue, postural dizziness, blood pressure, heart rate variation, and Raynaud's phenomenon. Six months after treatment initiation, she continued to experience fatigue, blood pressure variation, and dizziness. She also reported occasional leg swelling, most noticeable when standing still for prolonged periods. She noted an improvement in her ability to fulfill activities of daily living and in the severity of her Raynaud's phenomenon, which she attributed to LDN.

Demographics, medical history, and outcomes

Six patients who were initiated on LDN after having repeatedly failed other pharmacological treatments for POTS were reviewed. While half of the patients (3/6) noted an improvement in some of their symptoms, two discontinued the therapy due to a lack of noticeable improvement and one did not have documentation of any subjective change due to LDN. No patients reported significant side effects or adverse outcomes. Detailed descriptions of demographics, medical histories, and outcomes for each patient can be visualized in Table 2.

**Table 2:** Demographics, medical history, and outcomes. *Indicates score was collected ±2 months from respective visit time. PROMIS Fatigue: Patient-Reported Outcomes Measurement Information System Fatigue 10a; PROMIS Physical Health: Patient-Reported Outcomes Measurement Information System Physical Health V1.0; PROMIS Mental Health: Patient-Reported Outcomes Measurement Information System Mental Health V1.0; GAD-7: Generalized Anxiety Disorder Assessment; PHQ-9: Patient Health Questionnaire; COMPASS: Composite Autonomic Symptom Score

Patient	A	B	C	D	E	F
Age (years)	31	54	48	51	32	62
Racial identity	White	White	Black or African American	White	White	White
Biological sex	Female	Female	Female	Female	Female	Female
Insurance type	Medicaid	Private insurance	Private insurance	Private insurance	Private insurance	Private insurance
Preceding events prior to POTS symptom onset	COVID-19	None	None	GI viral illness	None	Surgery
Co-morbidities	ADHD, binge-eating disorder, bipolar II disorder, hypertension, PTSD, sensorineural hearing loss, migraine	Anxiety, arthritis, asthma, depression, hypertension, GERD, lymphocytic colitis, OSA, formerly obese (gastric bypass), insomnia, migraine, restless leg	Endometriosis, hypertension, IBS, pelvic floor dysfunction, fibromyalgia	Fibromyalgia, Hashimoto's thyroiditis, anxiety	Fibromyalgia, polyarthralgia, cognitive impairment	Raynaud's phenomenon, Sjogren's syndrome, degenerative disc disease, osteoporosis, limited scleroderma, GERD
Mean supine heart rate on tilt table	72	64	89	79	94	65
Max heart rate in the first 10 minutes of tilt table	119	97	126	138	134	142
Tilt table heart rate difference	47	33	37	59	40	77
QSART results	N/A	Abnormal	Yes	No abnormalities	N/A	Abnormal
Skin biopsy results	No abnormalities	Abnormal	N/A	No abnormalities	N/A	Abnormal
Days between initial and end visit	221	132	220	198	148	228
Starting dose of LDN	1 mg	1 mg	1 mg	1 mg	0.25 mg	1 mg
Dose 2 (time since start)	3 mg (4 months)	2 mg (2 months)	N/A	2 mg (4 months)	1 mg (1 month)	2 mg (2 months)
Dose 3 (time since start)	4 mg (5 months)	Discontinued (4 months)	N/A	N/A	2 mg (4 months)	3 mg (11 months)
Dose 4 (time since start)	2 mg (7 months)	N/A	N/A	N/A	3 mg (7 months)	N/A
Dose 5 (time since start)	1 mg (7 months)	N/A	N/A	N/A	4 mg (13 months)	N/A
Dose 6 (time since start)	Discontinued (7 months)	N/A	N/A	N/A	N/A	N/A
Medications at first visit	Brexpiprazole, clonazepam, esomeprazole, propranolol, gabapentin, lamotrigine, lisdexamfetamine dimesylate, lithium-carbonate, lorazepam, magnesium oxide, naratriptan, oral contraceptives, ondansetron, trazodone	Cetirizine, clonazepam, erenumab-aooe, ivabradine, magnesium oxide, modafinil, ondansetron, prednisone, pyridostigmine-bromide, rizatriptan-benzoate, tizanidine, trazodone, venlafaxine, zolmitriptan, zonisamide	Acebutolol, clobetasol, propionate, clonidine, famotidine, loratadine, lorazepam, ondansetron, tranexamic acid	Ivabradine, duloxetine, gabapentin, budesonide, famotidine, cetirizine, acetazolamide, pentoxifylline, mirtazapine, ketotifen-fumarate, losartan, bupropion, clonazepam, levothyroxine, medical marijuana	Ivabradine, prednisone, melatonin, cetirizine, midodrine, loratadine	Pyridostigmine, gabapentin, modafinil, oxycodone, celecoxib, cyclobenzaprine, denosumab, diazepam, diphenoxylate/atropine, estrogen, ipratropium, omeprazole, ondansetron, sennosides, simethicone, sucralfate, trazodone
Reasoning for starting LDN	Continued POTS symptoms, multiple treatment failures, neuropathic pain (failed 3 drugs for pain: gabapentin, tizanidine, Flexeril)	Pain	Continued POTS symptoms, repeated treatment failures, pain	Pain (gabapentin and increasing dose of duloxetine not sufficient to control)	Continued POTS symptoms, multiple treatment failures, neuropathic pain	Continued POTS symptoms, multiple treatment failures, Raynaud's phenomenon
Did the patient continue therapy for a full 6 months?	No	No	Yes	Yes	Yes	Yes
Why and when did the patient discontinue the therapy?	No improvement in pain	No improvement in pain	N/A	N/A	N/A	N/A
Did the patient report changes in symptoms after LDN?	No change	No change	Decreased	Decreased	Not charted	Decreased
PROMIS fatigue T- score initial*	76	N/A	N/A	N/A	67	55
PROMIS fatigue T- score end*	74	N/A	N/A	66	74	53
PROMIS-GHPH T-score initial*	29.6	34.9	34.9	32.4	34.9	47.7
PROMIS-GHPH T-score end*	23.5	34.9	26.7	29.6	32.4	47.7
PROMIS-GHMH T- score initial*	38.8	36.3	38.8	28.4	38.8	45.8
PROMIS- GHMH T-score end*	28.4	28.4	38.8	28.4	36.3	50.8
GAD-7 initial*	6	11	2	N/A	6	N/A
GAD-7 end*	7	12	4	N/A	3	2
PHQ-9 initial*	16	13	6	14	9	N/A
PHQ-9 end*	20	15	9	7	12	5
COMPASS total initial*	41.5	78.16	43.46	N/A	45.76	N/A
COMPASS total end*	N/A	71.3	47	N/A	57	66.98

**Figure 1:**
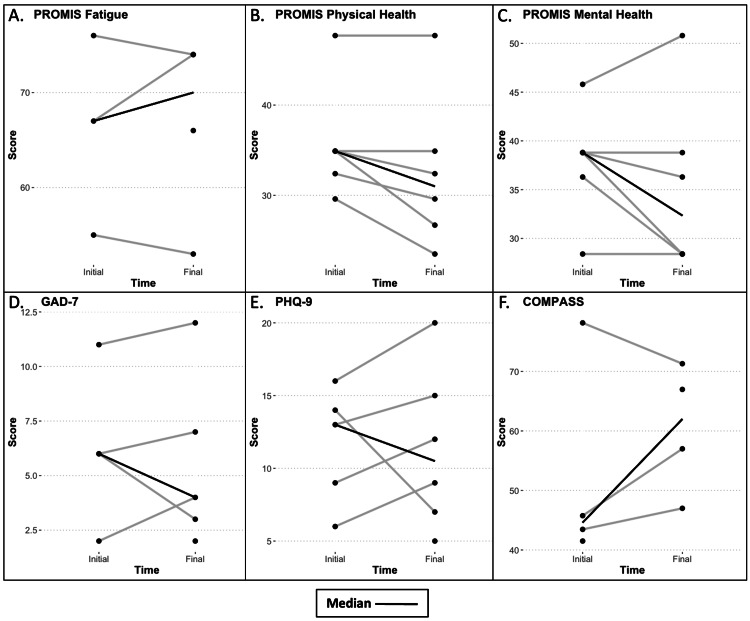
Patient-entered questionnaires. Scores at the initial time point were taken from the closest completed questionnaire ±2 months from LDN initiation. Scores at the final time point were taken from the closest completed questionnaire ±2 months from the 6-12 month visit. Single dots without a line represent patient scores that were only available at a single time point. The images show (A) PROMIS Fatigue: Patient-Reported Outcomes Measurement Information System Fatigue 10a; (B) PROMIS Physical Health: Patient-Reported Outcomes Measurement Information System Physical Health V1.0; (C) PROMIS Mental Health: Patient-Reported Outcomes Measurement Information System Mental Health V1.0; (D) GAD-7: Generalized Anxiety Disorder Assessment; (E) PHQ-9: Patient Health Questionnaire; (F) COMPASS: Composite Autonomic Symptom Score

Changes in patient-reported scales from initiation of therapy to the first follow-up six to 12 months after initiation were variable and can be visualized in Figures 1A-1F. Three patients completed the PROMIS fatigue questionnaire at both time points and two patients experienced decreases in fatigue levels. All patients completed the PROMIS Global Physical and Mental Health questionnaires. The median score of both measures decreased after LDN therapy, with only one patient having improvement in either measure. Interestingly, the median score on the GAD-7 and PHQ-9 decreased after therapy. Although the median scores for both questionnaires decreased, the majority of patients who completed these questionnaires at both time points saw increases in their individual scores, representing worsened anxiety and depression. Only three patients completed both time points of the COMPASS 31, with two showing an increase in scores indicating worsening autonomic symptoms and one showing a decrease in symptoms. No conclusions can be drawn from these findings due to the small sample size of our cohort and the differential response rate for these measures. However, these findings may indicate a variable response to LDN therapy in POTS and the need for tools and further studies to identify potential responders.

## Discussion

The exact mechanism by which LDN could exert a therapeutic effect in POTS is not yet certain. LDN is thought to work as a toll-like receptor 4 antagonist, leading to the modulation of glial activity [8,18,19]. LDN’s antagonism of toll-like receptor 4 inhibits downstream pathways that lead to the production of inflammatory cytokines, such as tumor necrosis factor (TNF)-a and interferon-b [18,20-22]. It has been demonstrated that LDN induces a metabolic shift in microglial cells from a pro-inflammatory to a quiescent anti-inflammatory phenotype and thus acts as an immunometabolic modulator that may decrease central sensitization [9,20]. This is in large part a basis for the trial of LDN in neuroinflammatory conditions and may in part explain the subjective improvement seen in some POTS patients. There is a growing body of evidence that autoimmunity and inflammation may play a role in the pathogenesis of POTS in a large number of cases. This has led to increased calls for clinical trials exploring immunomodulating therapies such as LDN [23-25].

Additionally, LDN may also exert beneficial effects on pain pathways. Through transient opioid receptor blockage in low doses, LDN can paradoxically lead to more opioid signaling and sensitivity. Increases in the endogenous opioid system may in turn lead to pain reduction and neuropsychiatric benefits [18,26]. Chronic pain is commonly seen in POTS patients, and subjective improvement may be linked to LDN’s impact on the opioid system [25,27].

This case series has several limitations, and its results should be interpreted with caution. Our sample size of six limited our ability to perform more advanced statistical analysis. In addition, due to the retrospective nature of the study, several outcome measures were unavailable for some patients. All patients in this study were taking additional pharmacological therapies for their POTS and thus we cannot say for certain whether the changes seen in outcome measures were due to the initiation of LDN alone, the actions of other therapies, or a combination thereof. We feel that despite these limitations, the strength of this work lies in our detailed descriptions of the treatment courses, which adds to the literature surrounding LDN use for POTS that previously consisted of a single case report [11].

Although LDN carries the mechanistic potential to impact POTS, there continues to be a lack of high-quality evidence that this transfers to functional outcomes. In our case series, there was a wide range of responses to LDN, and we were limited by our sample size and the inconsistency of dosing, follow-up, and patient-reported outcome measure completion. The lack of completed high-quality clinical trials of therapeutic options in POTS is not limited to LDN. The creation of such trials should remain a high priority for research as this growing patient population continues to wait for highly studied evidence-based therapies [6].

## Conclusions

While some patients noted subjective improvement with LDN, others expressed no improvement. All patients had variable changes in patient-reported outcome measures with the majority showing worsening of these measures after therapy. These findings may indicate a variable response to LDN therapy in POTS and the need for tools and further studies to identify potential responders. Our study was limited by the small sample size and incomplete collected outcome measures. Despite the lack of data on efficacy, our case series adds to the literature showing the safety and tolerability of the drug. However, we cannot yet recommend routine use. A large, randomized, double-blinded, placebo-controlled clinical trial is needed to fully assess LDN as a potential therapy for POTS.
